# Effects of Hydration on the Mechanical Properties of Salt-Doped Poly(methyl methacrylate)

**DOI:** 10.3390/molecules30122568

**Published:** 2025-06-12

**Authors:** Asae Ito, Naoki Uchida, Yusuke Hiejima, Koh-hei Nitta

**Affiliations:** Institute of Science and Engineering, Kanazawa University, Kakuma Campus, Kanazawa 920-1192, Japan; asae@se.kanazawa-u.ac.jp (A.I.); naoki991124@stu.kanazawa-u.ac.jp (N.U.); hiejima@se.kanazawa-u.ac.jp (Y.H.)

**Keywords:** poly(methyl methacrylate), mechanical properties, hygroscopicity, metal salts, nonequilibrium constitutive equation

## Abstract

The mechanical performance of poly(methyl methacrylate) (PMMA) is highly sensitive to moisture absorption, which induces plasticization and softening. In this study, we investigated the ductilization mechanism of PMMA by incorporating various metal salts with different cations (Li^+^ and Mg^2+^) and controlling water absorption through hygroscopic interactions. A nonequilibrium constitutive model is introduced, in which localized water domains around salt-rich regions gradually diffuse into the PMMA matrix during tensile deformation. The stress–strain behavior is described by combining rigid (dry) and soft (hydrated) matrix components, connected through an internal kinetic variable governed by the strain-dependent diffusion rate. The model successfully reproduces experimental tensile data and captures the transition from brittle to ductile behavior as a function of the moisture content. Notably, Mg salts exhibit stronger water binding and slower moisture redistribution than Li salts, resulting in distinct mechanical responses. These findings provide a mechanistic framework for tailoring the ductility of hygroscopic polymer systems via ion–water–polymer interactions.

## 1. Introduction

The physical properties of most polymeric materials are highly sensitive to environmental conditions, which poses significant challenges for their practical applications. In particular, the presence of moisture can induce plasticization and softening of polymeric materials owing to water absorption [1], making this a major concern for commodity plastics, excluding polyolefins, which exhibit relatively low moisture affinity. Because it is practically impossible to achieve complete moisture removal from solid-state polymers, environmental deterioration owing to water absorption must be taken into account for practical applications. A pragmatic strategy for mitigating this issue is to regulate the absorbed moisture content in the polymer, thereby achieving the desired mechanical performance. To this end, systematic investigations into the effects of water absorption on polymer mechanics are essential. However, maintaining and controlling a fixed moisture content within water-absorbed polymer solids remain technically challenging. In previous studies [2], we successfully controlled the water uptake of glassy poly(methyl methacrylate) (PMMA) by leveraging the strong water affinity of lithium through the addition of lithium salts to the polymer. In this study, we use this system to investigate the mechanism by which water absorption enhances the drawability of glassy polymers during the stretching process.

PMMA is a typical glassy polymer that is well known for its outstanding optical transparency among commodity plastics. It is widely used in applications such as optical lenses and aquariums, and it even shows potential for automotive glazing. However, the polar ester groups within PMMA readily form hydrogen bonds with water molecules [3]. This makes PMMA susceptible to significant reductions in stiffness and glass transition temperature upon moisture uptake, resulting in pronounced plasticization and softening effects. Numerous studies have investigated the effect of environmental humidity on the mechanical properties of PMMA, including its tensile properties [4,5,6,7,8], scratch resistance [9], crack propagation behavior [10,11,12], fracture characteristics [13], and fatigue resistance [14]. These studies collectively highlight the instability of the mechanical performance of PMMA under varying moisture conditions [15]. Nevertheless, from an application-oriented perspective, the moisture sensitivity of PMMA could be leveraged to overcome its intrinsic brittleness, thereby broadening its functional versatility.

In general, the incorporation of metal salts in polymer matrices has been extensively reported as a strategy for inducing ionic cross-linking, which modulates the mechanical and viscoelastic behavior of ionomers. For example, Yoshikawa et al. [16] demonstrated that the viscosity of ionized polystyrene/polyamide 6 blends is influenced by the type of ionic species. Takahashi et al. [17] showed that the elongational viscosity in poly(ethylene-co-methacrylic acid) ionomers is enhanced in the presence of ionic interactions with added metal salts. Storey and co-workers [18] reported that metal salts can act as ionic plasticizers in ionomer systems. Miyagawa et al. [14] extended this approach to glassy polymers, showing that doping PMMA with LiCF_3_SO_3_ increases its glass transition temperature via ion–dipole interactions while preserving high transparency. In previous works, we systematically studied the effects of incorporating various lithium salts—including LiCF_3_SO_3_, LiC_4_F_9_SO_3_, LiClO_4_, LiN(CF_3_SO_2_)_2_, LiBr, LiCl, and LiI—in PMMA. These salts were found to associatively interact with the polymer chains and to finely disperse within the PMMA matrix as “pinning sites”, leading to an increase in the glass transition temperature [15,19,20,21,22,23]. Furthermore, owing to their high hygroscopicity, these salts concentrate absorbed water in their vicinity, enabling control of the water content—and consequently the mechanical properties—of PMMA by adjusting the type and concentration of the salt. We have also reported that the water absorption resulting from the addition of salts leads to the plasticization of PMMA.

The aim of this study is to investigate the tensile properties of PMMA samples with different moisture content levels and to elucidate the mechanism underlying the changes in the stress–strain behavior induced by water absorption. For this purpose, we applied a nonequilibrium constitutive model in which the water domains surrounding salt-rich local domains diffuse into the dried PMMA matrix during stretching to PMMA samples doped with salts composed of different cationic and anionic species.

Understanding and controlling such moisture-responsive behavior not only provide insights into the fundamental mechanics of hydrated polymers but also open up avenues for practical applications. In particular, this behavior is promising for applications in wearable sensors [24], where controlled ductility and environmental sensitivity are advantageous.

## 2. Experiments

### 2.1. Sample Preparation

PMMA pellets with a weight-average molecular weight (*M*_w_) of 1.0 × 10^5^ and a polydispersity index (*M*_w_/*M*_n_) of 1.9 were used in this study. These values were determined by gel permeation chromatography using a PMMA standard (EasiVial PM, product nos. PL2020-0201 and PL2020-0202; Agilent Technologies California U.S., Ltd., Tokyo, Japan). The PMMA pellets and a metal salt, such as LiClO_4_ (NACALAI TESQUE, INC., Kyoto, Japan) or Mg(ClO_4_)_2_ (KISHIDA CHEMICAL CO., LTD., Osaka, Japan), were dissolved in a mixed solvent of dichloromethane and methanol (weight ratio 9:1) and stirred for 1 h. The solution was then air-dried at room temperature under a fume hood for 24 h, followed by vacuum-drying at 135 °C for 30 h to remove the residual solvents and obtain cast films. According to our previous study [25], the dynamic mechanical properties had no change between these dried sheets and the compression-molded sheets, indicating that no plasticization due to residual solvent occurred. We also confirmed by gas chromatography that vacuum heat-drying effectively reduced the residual solvent content to below 0.1% [25]. The resulting films were pulverized and compression-molded at 200 °C and 20 MPa using a benchtop hot press, followed by rapid quenching at 25 °C to form measurement sheets with a thickness of approximately 200 μm. The molar ratios and weight fractions of the salts used in the solution-cast films are summarized in Table 1.

### 2.2. Determination of the Water Contents in the Samples

Water-absorbed samples were prepared by storing the sheets in a humidity-controlled chamber. The relative humidity in the chamber was maintained at 70% at 25 °C using the saturated salt method. The sheets with varying moisture contents were prepared by storing them in the chamber for different time intervals. The water content in each sample was measured by the Karl Fischer coulometric titration method using a Karl Fischer oven (220–230 °C, standard moisture content 5.55%; Honeywell, Charlotte, NC, USA) and a Metrohm 899 coulometer (Metrohm, Tokyo, Japan) at 230 °C.

### 2.3. Infrared Spectroscopy

To investigate the molecular interactions induced by the addition of metal salts from the perspective of molecular vibrations, we performed Fourier transform infrared (FT-IR) spectroscopy using a Nicolet iS5 spectrometer (Thermo Fisher Scientific, Waltham, MA, USA) equipped with an attenuated total reflectance accessory. The spectra were recorded at a resolution of 2 cm^−1^ with 36 accumulated scan times.

### 2.4. Tensile Tests

The uniaxial tensile tests were performed using a compact tensile tester (Abe Seisakusho, Nagaoka, Japan). Rectangular specimens (width of 5 mm, gauge length of 10 mm) were used. The tests were performed at 25 °C with a fixed crosshead speed of 10 mm min^−1^. During the tensile tests, a relative humidity of 70% was maintained by operating multiple humidifiers simultaneously.

## 3. Theoretical Framework

Here, we introduce a nonequilibrium constitutive model to describe the effects of moisture on the stress–strain behavior of salt-doped PMMA. Under the initial conditions, water-rich regions (hydrated domains) are assumed to form around salt-rich regions. As tensile deformation progresses, these hygroscopic regions are expected to gradually spread throughout the specimen via strain-induced diffusion. Taking this mechanism into account, we constructed a constitutive equation in which the dried or initial PMMA matrix is modeled as an incompressible, highly rigid material that undergoes brittle fracture, whereas the fully moisturized matrix in equilibrium is assumed to behave as a plastically deformable fluid.

According to Leonov [26], the constitutive relation for such incompressible rigid materials can be derived from a strain energy density function $W_{D}$ based on the second strain invariant:

(1) $$W_{D}=\frac{1}{2}G_{D}(2\lambda +\lambda^{-2}-3)$$

where λ is the extension ratio and $G_{D}$ is the rigidity. Consequently, the constitutive relation for the initial salt-doped PMMA can be derived using $dW_{D}/d\lambda$ as follows:

(2) $$\sigma_{D}=G_{D}(1-\lambda^{-3})$$

Therefore, Young’s modulus of the initial PMMA/salt sample is given by 3*G_D_*.

In contrast, the fully moisturized matrix (soft component) undergoes plastic deformation at a constant stress level $\sigma_{p}$. Therefore, its stress–extension relationship can be reasonably approximated by a step function, $\Theta \left[x\right]\;(x\in R)$. Accordingly, the constitutive equation for water-saturated PMMA/salt samples can be expressed as

(3) $$\sigma_{H}=\sigma_{p}\theta [\lambda -1]$$

The value of $\sigma_{p}$ for each water-absorbed sample corresponds to the stress level or strength of the ductile region and decreases with increasing water content.

Here, we introduce the internal variable $\alpha \;\left(0\leq \alpha \leq 1\right)$ to describe the nonequilibrium state in which the moisture surrounding the salt domains gradually diffuses into the PMMA matrix as the tensile deformation proceeds. The internal variable α represents the stress contribution from the dried matrix region; in other words, 1−α corresponds to the contribution of the hygroscopic regions relative to the dried matrix. On the basis of the strategy in the Leonov model [26], the simple constitutive relation for the moisturized sample can be expressed using parameter *α* as follows

(4) $$\sigma =\alpha \sigma_{D}+(1-\alpha )\sigma_{H}$$

The spreading process of the hygroscopic regions around the salt over the entire sample specimen can be described using the strain dependence of the internal variable α. The *α* value represents the fraction of the dry component $\sigma_{D}$ and serves as a kinetic transfer parameter. As the hygroscopic regions grow during stretching, the fraction of the dry matrix accordingly decreases. Thus, the fraction α is reduced by the strain, reflecting the progressive release of water from the hygroscopic domains. The transfer process during extension is simply assumed to be the first-order approximate kinetics with respect to the current strain, where the driving force for the transfer process is assumed to be proportional to the internal variable:

(5) $$\frac{d\alpha }{d\ln\lambda }=-k\alpha$$

Here, the rate constant *k* corresponds to the diffusion rate of water within the hygroscopic regions around the salt in the dried PMMA matrix. The driving force for water diffusion may result from Poisson shrinkage in the direction perpendicular to the axis of elongation.

At the initial stage, the water pools are confined within the salt-rich domains, indicating that *α* = 1 at *λ* = 1. By integrating Equation (5) under the initial condition, α=1 at λ=1, we obtained the following equation:

(6) $$\alpha =\lambda^{-k}$$

indicating that the fraction *α* exponentially decays with increasing extension. Consequently, by substituting Equations (2), (3), and (6) into Equation (4), we have the following nonequilibrium constitutive equation describing the spreading of the hygroscopic regions in the dried matrix:

(7) $$\sigma =\lambda^{-k}G_{D}(1-\lambda^{-3})+(1-\lambda^{-k})\sigma_{p}\theta [\lambda -1]$$

This is a kinetically constitutive relation for describing the gradual change from brittle to ductile behavior during extension.

## 4. Results and Discussion

**Figure 1:**
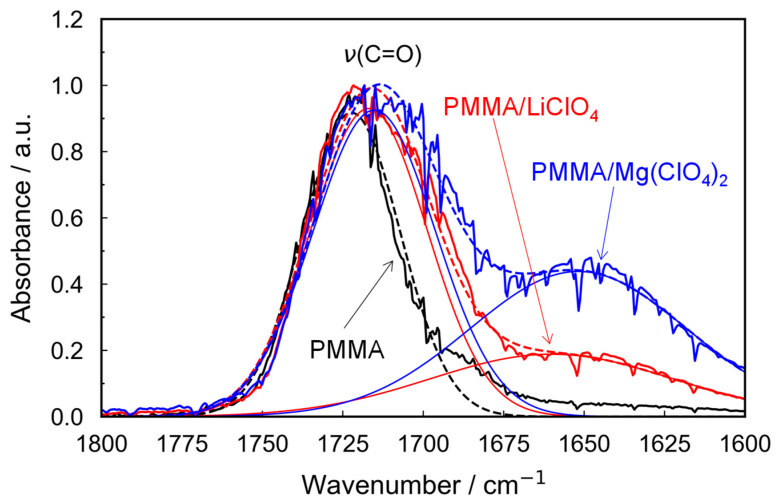
Fourier transform infrared spectra of PMMA, PMMA/LiClO_4_, and PMMA/Mg(ClO_4_)_2_. Molar concentration of both salts is 0.11. The solid line represents experimental data, and the dotted line represents the fitting curve.

We compared the carbonyl absorption bands in the FT-IR spectra of the pristine PMMA and metal salt-doped PMMA samples (Figure 1). The peak attributed to the carbonyl stretching vibration at 1720 cm^−1^ extended toward lower wavenumbers. The red shift in the 1720 cm^−1^ band indicates that the carbonyl groups experienced a stretching force owing to interactions with metal cations, suggesting stronger interactions in the case of Mg salt compared with those with monovalent Li salt. In addition, the salt-doped PMMA samples exhibited a novel broad peak at 1650 cm^−1^ (the bending vibration mode of the hydroxy group), which is associated with water absorption. These results suggest that the divalent Mg^2+^ ion interacts more strongly with water molecules than the monovalent Li^+^ ion at the same molar ratio. The peak area around 1650 cm^−1^ in the PMMA/Mg-salt system, as obtained from peak deconvolution analysis (see Figure 1), was nearly twice that of the PMMA/Li-salt system. In the PMMA/Mg-salt system, the peak area observed at approximately 1650 cm^−1^ using peak deconvolution (Figure 1) was nearly twice that of the PMMA/Li-salt system. This result suggests that Mg^2+^ ions promote greater water absorption than Li^+^ ions, proportion to their cation valence at the same molar ratio, and further indicates that both salts are dispersed as dissociated ions within the PMMA matrix.

**Figure 2:**
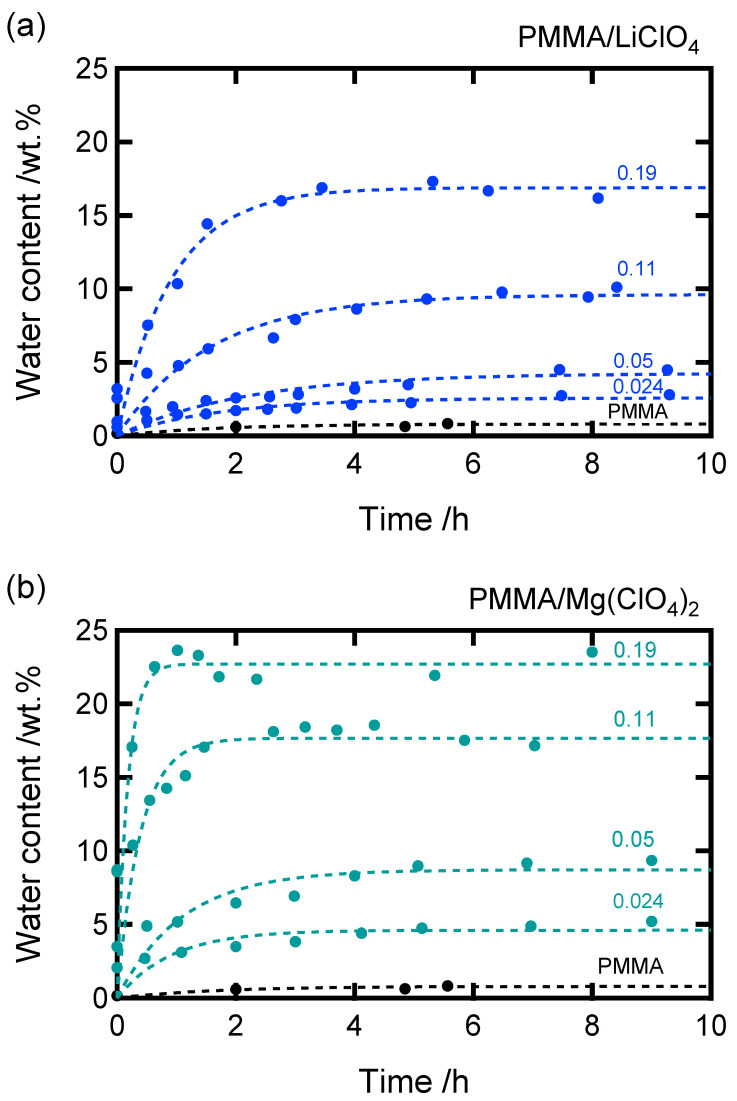
Changes in water absorption with time for the (**a**) PMMA/LiClO_4_ and (**b**) PMMA/Mg(ClO_4_)_2_ samples. The solid symbols represent the experimental data, and the dashed lines represent the theoretical curves calculated by Equation (8).

The time-dependent water absorption behavior of the samples is shown in Figure 2. For both the Li- and Mg-doped systems, the water content increased with time and eventually reached a plateau, indicating water saturation. Assuming pseudo-first-order kinetics for water absorption, we can express the following equation representing the time dependence of the water concentration:

(8) $$c_{w}=c_{\infty }(1-e^{-t/\tau })$$

where $c_{w}$ is the water concentration, $c_{\infty }$ is the equilibrium concentration at t→∞, and *τ* is the inverse of the absorption rate. The theoretical curve derived from Equation (8) showed good agreement with the experimental data, as shown in Figure 2. The curve fitting yielded the value of $c_{\infty }$.

**Figure 3:**
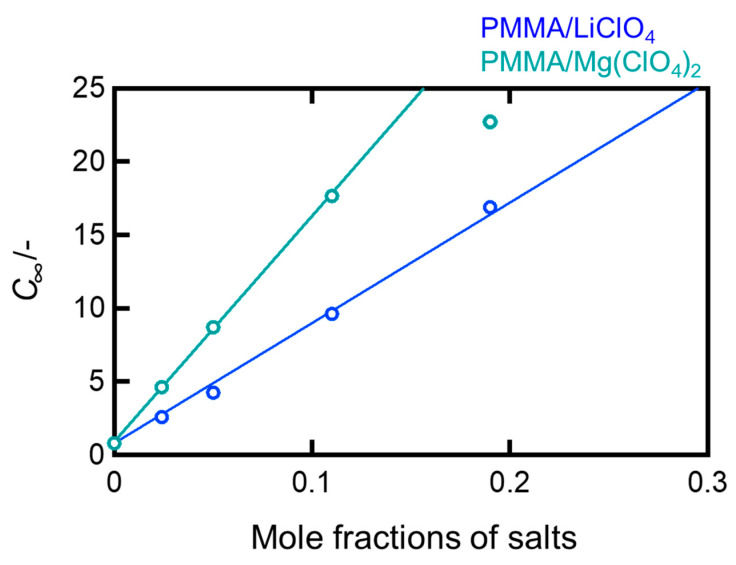
Mole fraction of salts plotted against the equilibrium coefficient of water absorption *c*_∞_ for PMMA/LiClO_4_ and PMMA/Mg(ClO_4_)_2_.

For the PMMA samples dopped with LiClO_4_ and Mg(ClO_4_)_2_, the equilibrium concentration $c_{\infty }$ is plotted against the molar fraction of the salt in Figure 3. There was a linear relationship between $c_{\infty }$ and the molar fraction of the salt. The regression lines were plotted excluding the PMMA/Mg(ClO_4_)_2_ (0.19) sample, which underwent deliquescence owing to an excessively high salt concentration. It is interesting to note that the slope of the curve for the Mg salt systems was approximately twice that for the Li salt systems, indicating that the divalent Mg^2+^ ion induces roughly two times more water absorption than the monovalent Li^+^ ion at the same molar ratio. As previously described, this also suggests that the salts likely dispersed as individual ions or small clusters in the PMMA matrix, rather than aggregating to form clusters.

We also investigated the tensile behavior of the salt-doped PMMA samples. The effects of moisture on the stress–strain curves for both systems are summarized in the Supplementary Materials. Completely dry conditions were difficult to achieve, and even the dried samples retained a few percent moisture content. Both the PMMA/LiClO_4_ (0.40) and PMMA/Mg(ClO_4_)_2_ (0.19) samples exhibited a brittle-to-ductile transition above 16% moisture content. The low moisture PMMA/salts used in this study exhibited brittle fracture behavior that was nearly independent of the salt content (see Figure S1). The PMMA/LiClO_4_ (0.11) system, containing only a small amount of salt, showed no ductilization regardless of the water content, confirming that the addition of salt led to embrittlement. Videos of the tensile deformation of dry PMMA, as well as both water-saturated PMMA/salts, are provided in the Supporting Information (see Videos S1–S3).

**Figure 4:**
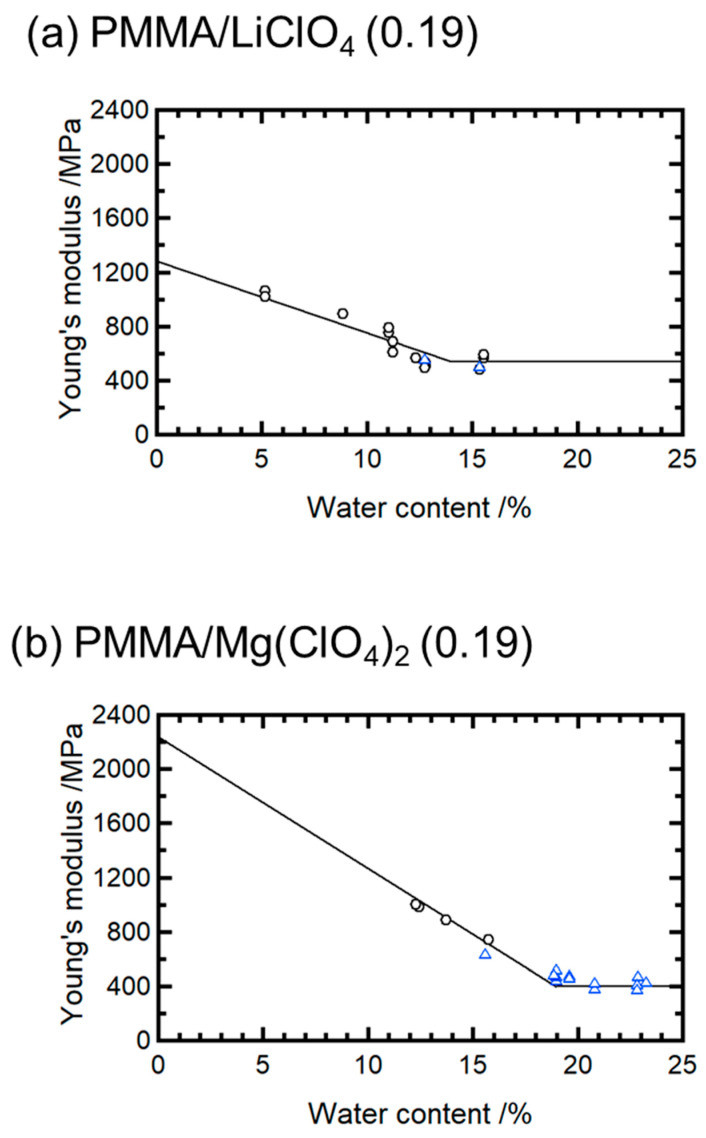
Young’s modulus of dried and water-absorbed (**a**) PMMA/LiClO_4_ (0.19) and (**b**) PMMA/Mg(ClO_4_)_2_ (0.19). The circular symbol represents Young’s modulus value obtained from the stress–strain curves of brittle samples, and the blue triangular symbol represents Young’s modulus value obtained from the stress–strain curves of ductile samples.

The dependence of Young’s modulus on the water content for PMMA/LiClO_4_ (0.19) and PMMA/Mg(ClO_4_)_2_ (0.19) is shown in Figure 4. Although small amounts of residual moisture remained after drying, their influence was consistent across samples and did not affect the relative trends observed in the ductility and modulus reduction. For both systems, the modulus decreased linearly with increasing the water content in the brittle state but plateaued after the transition to the ductile state. The slopes of the linear regions indicate that the modulus declines per 1% moisture increase, 53 MPa/% for Li^+^ and 97 MPa/% for Mg^2+^, suggesting a twofold greater reduction for Mg^2+^ compared with that for Li^+^.

In a previous study, we found that the embrittlement caused by the addition of salt is governed by the average relaxation time of the melt flow [15]. Ion aggregation along the PMMA chains forms structural defects, degrading the toughness. The intercept of the extrapolated modulus in Figure 4 represents the dry-state modulus. This value was approximately two times higher for the Mg systems than for the Li systems, indicating the stronger pinning effect of Mg^2+^. Consistently, the stronger embrittlement observed in Mg systems is attributed to enhanced ionic pinning interactions along the PMMA backbone, which suppress molecular mobility and increase structural rigidity.

**Figure 5:**
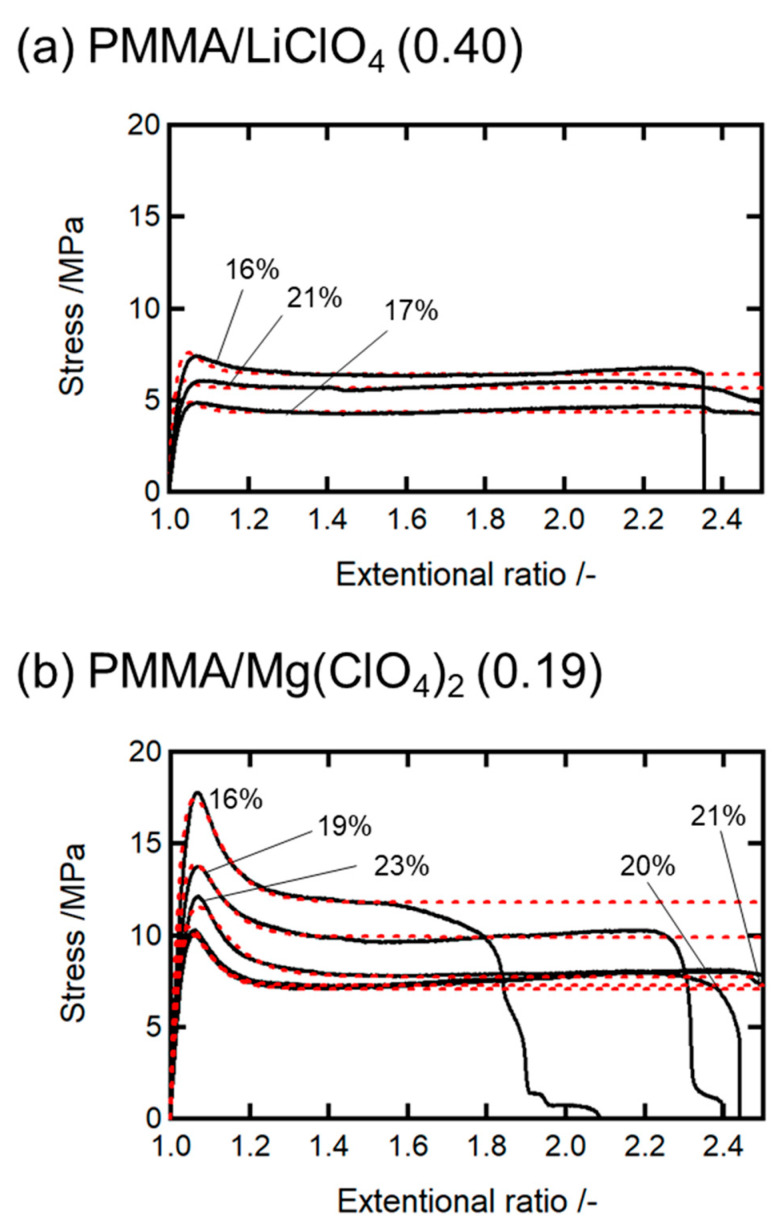
Results of fitting analysis of the stress–strain curves for (**a**) PMMA/LiClO_4_ (0.40) and (**b**) PMMA/Mg(ClO_4_)_2_ (0.19).

Here, we analyze the ductilization mechanism of the water-absorbed PMMA/salt systems based on the nonequilibrium constitutive formulation. The stress values in the ductile region relaxed at a constant level after exhibiting a maximum or yield peak, and the equilibrium level decreased with increasing water content (see Figure 5). We consider that the water-rich domains, which were initially localized around the salt particles, gradually spread throughout the polymer matrix during tensile deformation. This redistribution process was modeled kinetically, because it led to a homogeneous water-absorbed state in the specimen, allowing for plastic deformation. We found that the constitutive equation (Equation (7)) reproduced the experimental stress–strain curves well for both the 20 and 30 wt.% salt-doped samples, as shown in Figure 5a,b. The parameter *G_D_* can be determined from the *E_Y_* values using $E_{Y}=3G_{D}$. The parameter $\sigma_{p}$ was also determined using the average values of the fracture stress.

**Figure 6:**
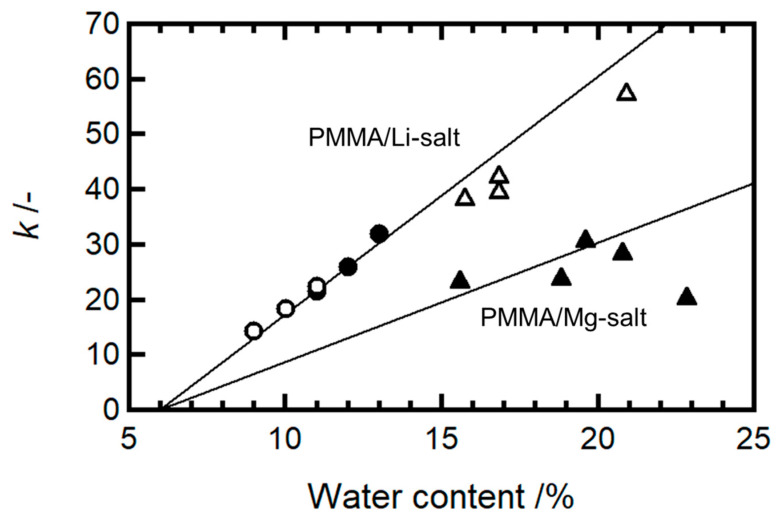
Kinetic parameter (*k*) of PMMA/LiCF_3_SO_3_ (0.16) (open circle), PMMA/LiCF_3_SO_3_ (0.28) (closed circle), PMMA/LiClO_4_ (0.40) (open triangle), and PMMA/Mg(ClO_4_)_2_ (0.19) (closed triangle) as a function of the water content. The first two datasets have already been reported in our previous publication [2].

The stress–strain curves for both systems were sufficiently well fitted by adjusting the parameter *k*. The rate constant *k* was obtained from the fitting curves shown in Figure 5. The values of Young’s modulus, the plastic flow stress $\sigma_{p}$, and the kinetic parameter *k* values are listed in Table 2. The *k* values were then plotted as a function of the water content for both systems (Figure 6). In the figure, the data of PMMA/LiCF3SO3 (0.16) and PMMA/LiCF3SO3 (0.28) previously published [2] were included. The slope of *k* for the Mg-salt systems was approximately half that of the Li salt systems, indicating that the interaction between Mg^2+^ and water is twice as strong as that between Li^+^ and water. Consequently, water diffusion is more suppressed in the Mg-salt-doped PMMA, indicating more limited redistribution of the moisture during deformation. This finding suggests that the kinetic parameter *k*, which reflects water diffusion, is mainly governed by the cation species, and it is largely unaffected by the anion species.

## 5. Conclusions

In this study, we developed a nonequilibrium constitutive equation to describe the mechanical behavior of water-absorbed PMMA doped with various metal salts. By considering the strain-induced diffusion of water from the hygroscopic regions around the salt domains, the model successfully reproduced the experimentally observed transition from brittle to ductile behavior. The internal kinetic variable introduced in the model captures the progressive redistribution of moisture during deformation, allowing for a unified description of both the dry and hydrated states.

The results revealed that the mechanical softening and ductilization of PMMA are strongly governed by the type and concentration of the metal salt. Divalent Mg^2+^ salts exhibited stronger interactions with water than monovalent Li^+^ salts, leading to higher water uptake but reduced mobility of absorbed water during deformation. This resulted in more pronounced embrittlement in the dry state and a slower transition to ductile behavior under moist conditions. The kinetic parameter *k*, representing moisture diffusion, was mainly influenced by the cation species, and it was less sensitive to the anion type. The proposed model is capable of extending to other hygroscopic polymer systems and serve as a predictive tool in the development of moisture-responsive materials.

These findings provide fundamental insights into the moisture-induced mechanical tuning of glassy polymers and offer a rational framework for designing polymer materials with tailored mechanical properties through ion–water–polymer interactions. We have previously pointed out that the molecular motion of hygroscopic domains induces molecular slippage and plastic flow of PMMA chains under tensile deformation in moisture-absorbed PMMA/salt systems [2]. Such hydration around salt ions may also influence interfacial lubrication properties, particularly under sliding or contact conditions. Recent studies [27] suggest that water enhances lubrication via hydration shells, which generate a hydration repulsive force capable of sustaining a large normal load and exhibiting a fluid response to shear between a hydrated silicon nitride ball and a sapphire substrate.

The results of this study indicate that the mechanical properties of polar glassy polymers, such as PMMA, can be effectively tailored by tuning the valence of the metal cation in the added salt. This framework provides a physical basis for interpreting the moisture-dependent mechanical behavior of PMMA-based systems, and it may be applicable to other polar glassy polymers subjected to humid environments.

## Figures and Tables

**Table 1:** Experimental conditions for the preparation of the solution-cast sheets of the PMMA/LiClO_4_ and PMMA/Mg(ClO_4_)_2_ samples with different molar ratios of the salt.

Sample Code	Mole Fraction of Salt/mol mol^−1^	Weight Fraction of Salt /wt.%
PMMA	0	0
PMMA/LiClO_4_ (0.02)	0.024	2.40
PMMA/LiClO_4_ (0.05)	0.050	5.00
PMMA/LiClO_4_ (0.11)	0.11	10.6
PMMA/LiClO_4_ (0.19)	0.19	17.0
PMMA/LiClO_4_ (0.40)	0.40	30.0
PMMA/Mg(ClO_4_)_2_ (0.02)	0.024	5.00
PMMA/Mg(ClO_4_)_2_ (0.05)	0.050	10.0
PMMA/Mg(ClO_4_)_2_ (0.11)	0.11	20.0
PMMA/Mg(ClO_4_)_2_ (0.19)	0.19	30.0

**Table 2:** Young’s modulus, plastic flow stress $\sigma_{p}$, and kinetic parameter *k* values.

Sample Code	Moisture Content /wt.%	Young’s Modulus/MPa	*k*/-	*σ*_p_/MPa
PMMA/LiClO_4_ (0.40)	16	302	38	6.4
PMMA/LiClO_4_ (0.40)	17	189	42	4.3
PMMA/LiClO_4_ (0.40)	21	267	57	5.6
PMMA/Mg(ClO_4_)_2_ (0.19)	16	628	23	12
PMMA/Mg(ClO_4_)_2_ (0.19)	19	480	23	9.9
PMMA/Mg(ClO_4_)_2_ (0.19)	20	472	30	7.2
PMMA/Mg(ClO_4_)_2_ (0.19)	21	416	28	7.0
PMMA/Mg(ClO_4_)_2_ (0.19)	23	369	20	7.7

## Data Availability

Data are contained within the article and Supplementary Materials.

## Supplementary Materials

The following supporting information can be downloaded at: https://www.mdpi.com/article/10.3390/molecules30122568/s1.
