# Supplementary material for: Effects of Hydration on the Mechanical Properties of Salt-Doped Poly(methyl methacrylate)
Source: Molecules. 2025 Jun 12;30(12):2568. doi: 10.3390/molecules30122568 (PMC12195638; doi:10.3390/molecules30122568)
Supplement: Supplementary file 1 [file molecules-30-02568-s001.zip › molecules-3636490-supplementary.pdf]

Supplementary material

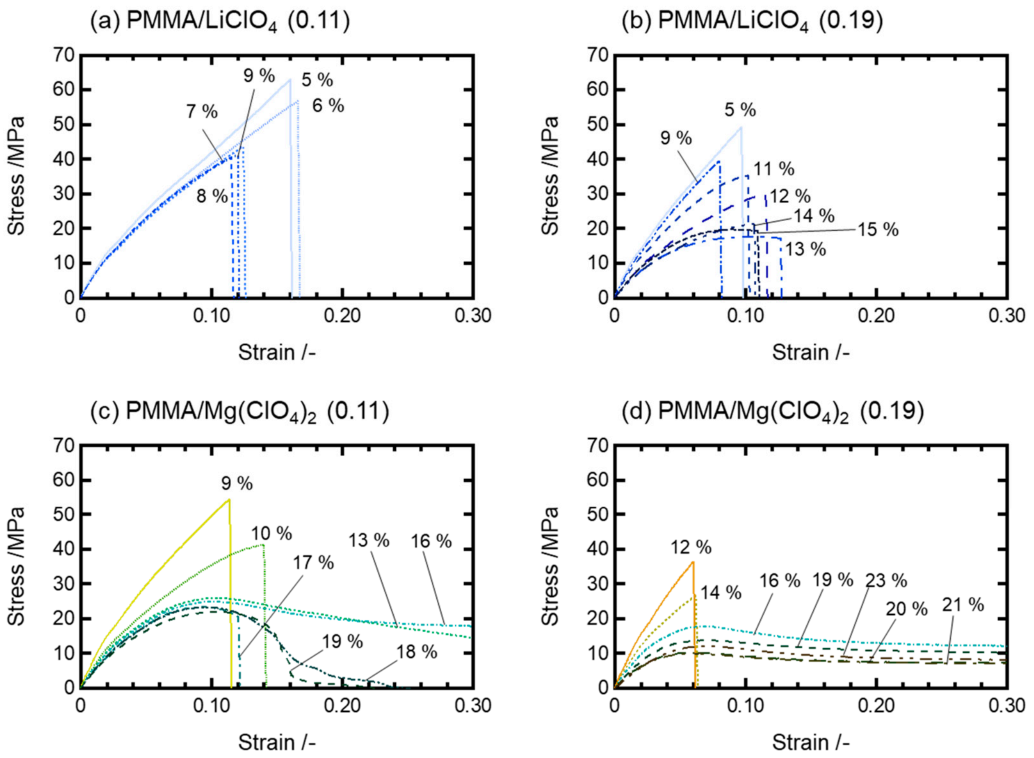

Figure S1 Overall stress–strain curves for each sample (a) PMMA/$LiClO_4$ (0.11), (b) PMMA/$LiClO_4$ (0.19), (c) PMMA/$Mg(ClO_4)_2$ (0.11) and (d) PMMA/$Mg(ClO_4)_2$ (0.19).

Video S1 Tensile deformation behavior of water-saturated PMMA.

Video S2 Tensile deformation behavior of water-saturated PMMA/$LiClO_4$ (0.40) under uniaxial stretching. The water content is 16 %.

Video S3 Tensile deformation behavior of water-saturated PMMA/$Mg(ClO_4)_2$ (0.19) under uniaxial stretching. The water content is 21 %.
